# Predictive nomogram of ultrasound indicators for the termination outcome of caesarean scar pregnancy

**DOI:** 10.1038/s41598-024-82894-7

**Published:** 2024-12-28

**Authors:** Xiaoyi Xiao, Zhichao Feng, Ting Li, Hong Qiao, Yun Zhu

**Affiliations:** 1 Department of Ultrasound, The First Hospital of Hunan University of Chinese Medicine, Hunan University of Chinese Medicine, Changsha, 410021 Hunan People’s Republic of China; 2 Department of Radiology, The Third Xiangya Hospital, Central South University, Changsha, 410013 Hunan People’s Republic of China; 3 Department of Medical Imaging, Yueyang Central Hospital, No. 39 Dongmaoling Road, Yueyang, 414020 Hunan China; 4 Department of Intensive Care Unit, Li County People’s Hospital, Changde City, Hunan China

**Keywords:** Nomogram, Caesarean scar pregnancy, Adverse event, LASSO, Diseases, Health care, Risk factors

## Abstract

To develop and validate a nomogram for predicting the risk of adverse events (intraoperative massive haemorrhage or retained products of conception) associated with the termination of Caesarean scar pregnancy (CSP). Data were retrospectively collected from patients diagnosed with CSP who underwent Dilation and Curettage (D&C) at two hospitals. This data was divided into internal and external cohorts for analysis. The internal cohort was randomly split, with 70% of the data designated for a training set and 30% for an internal validation set. The external cohort served exclusively as the external validation set. LASSO and logistic regression techniques were employed to select variables and construct the nomogram. The performance of the nomogram was evaluated using various methods, including C-index, calibration curve, decision curve analysis (DCA), and clinical impact curve analysis (CICA), to assess its identification, calibration, and clinical effectiveness. The prediction nomogram included several predictors, such as scar thickness, type of CSP, gestational sac diameter, and blood flow. It demonstrated strong discrimination, with a C-index of 0.83 (95% confidence interval: 0.77–0.89). Furthermore, in the internal validation set, a high C-index of 0.78 was achieved, while in the external validation set, it reached 0.83. Additional assessments using calibration curve analysis, DCA, and CICA indicated robust agreement between the nomogram’s predictions and actual observations, highlighting its utility and reliability. The developed nomogram shows excellent discriminative ability and calibration, with the potential for effective local prediction of adverse events in CSP.

**Supplementary Information:**

The online version contains supplementary material available at 10.1038/s41598-024-82894-7.

## What does this study adds to the clinical work

Our study introduces an innovative nomogram that integrates scar thickness, CSP type, gestational sac diameter, and blood flow parameters to accurately predict adverse events during the termination of CSP. Comprehensive validation in both internal and external cohorts demonstrates the nomogram’s robust discriminatory power (C-index = 0.83) and clinical efficacy, as confirmed by calibration, decision curve analysis, and impact curve analysis. This tool offers clinicians a valuable resource for the early identification of high-risk CSP patients, thereby facilitating personalized management strategies and ultimately enhancing patient outcomes.

## Introduction

Caesarean scar pregnancy (CSP), a rare type of ectopic pregnancy, refers to the implantation of the gestational sac at the scar site from a previous uterine incision^1^. In recent years, the incidence of CSP has significantly increased due to the implementation of China’s multiple birth policy, the rising rate of caesarean sections, and the widespread use of ultrasound diagnosis^2^.

CSP is linked with severe complications such as massive haemorrhage and uterine rupture. Therefore, upon diagnosis of CSP, it is crucial to promptly terminate the pregnancy and remove the gestational tissue as early as possible^3^. Because the safety and effectiveness of transvaginal surgery under ultrasound or hysteroscopic guidance for uterine evacuation are relatively good, it is now widely adopted in China. However, adverse event such as intraoperative massive haemorrhage and retained products of conception (RPOC) still cause high concern among clinical doctors^4,5^. Because intraoperative massive haemorrhage may lead to more serious maternal bleeding, shock, or even death, while postoperative RPOC may result in complications such as infection, uterine trauma, and infertility^6–8^. It is crucial to predict the occurrence of the adverse events through early indicators of CSP.Ultrasonography is a safe, non-invasive, and relatively inexpensive imaging modality, thus making it the preferred method for early diagnose of CSP ^9^. Through ultrasonography, doctors can thoroughly assess the uterine scar area, including scar thickness, the relationship between CSP and the scar, and parameters such as blood flow around the implant site. This non-invasive approach is important for early detection and diagnosis of CSP^10^.Therefore, if this study can construct a nomogram based on ultrasound indicators to predict the probability of adverse events during the management of CSP in the early time, it would provide more reliable evidence for clinical doctors to formulate more personalized treatment plans, thereby improving patient outcomes and survival rates.

## Patients and method

This study was approved by the Institutional Review Boards of Third Xiangya Hospital, Central South University, and the First Affiliated Hospital of Hunan University of Chinese Medicine and informed consent was obtained from all subjects or their legal guardian(s). The research adhered to the principles of the Helsinki Declaration.

### Patients

This study involved patients who were admitted to the Third Xiangya Hospital of Central South University from January 2015 to February 2024, diagnosed with CSP, and treated as an internal cohort. The diagnosis of CSP was based on the following indicators from the 2016 edition of the Expert Consensus on the Diagnosis and Treatment of Caesarean Scar Pregnancy after Caesarean Sect^11^, published by the Reproductive Health Group of the Obstetrics and Gynaecology Branch of the Chinese Medical Association: specifically, the patient’s history of caesarean section, history of menstrual cessation, human chorionic gonadotropin (hCG) levels, ultrasonography, and postoperative pathology. Clinical and ultrasound data of CSP patients were collected by two independent researchers from the hospital’s electronic medical record system and ultrasound department database, following specific inclusion and exclusion criteria. Inclusion criteria: (1) History of previous caesarean section; (2) Patients with elevated serum β-hCG; (3) Preoperative ultrasound diagnosis of CSP and ultrasound review results one month postoperatively; (4) Gestational age ≤ 12 weeks at the time of surgery and the interval between dilation and curettage (D&C) and ultrasound examination was less than 1 week; (5) Pathological results indicating pregnancy tissue. Exclusion criteria: (1) Termination of pregnancy was not done by D&C; (2) Prior treatments before D&C such as medications (e.g., methotrexate, ), uterine artery embolization (UAE), and high-frequency ultrasound ablation aimed at reducing blood supply to the gestational sac and intraoperative bleeding; (3) Cases of twin or multiple pregnancies; (4) Uterine malformations and anomalies; (5) Patients with bleeding disorders; (6) CSP patients who were misdiagnosed with intrauterine pregnancy and underwent artificial or medical abortion at other hospitals. The patients who met the selection criteria and were finally included were divided into two groups: the case group (those with intraoperative blood loss exceeding 200 ml^12,13^ or RPOC observed on postoperative ultrasound examination^14^) and the control group (those with intraoperative blood loss less than 50 ml and no RPOC observed on postoperative ultrasound examination). The included patients were randomly divided into a training set and an internal validation set in a 7:3 ratio. Additionally, according to the same inclusion criteria, patients hospitalized for CSP at the First Affiliated Hospital of Hunan University of Chinese Medicine from January 2016 to December 2023 were used as an external cohort, namely external validation set. We de-identified all patient details to protect their identity.

### Ultrasound diagnosis and classification of CSP

All ultrasound data relevant to our study were obtained at the time of CSP diagnosis, prior to initiating treatment. The ultrasonographic criteria used for diagnosing CSP included the following^3^: (a) The gestational sac is developing in the front part of the lower portion of the uterus. (b) The uterus and cervical canal appear empty. (c) There is no healthy muscular wall (myometrium) between the gestational sac and the bladder. According to the relationship between the gestational sac and the caesarean section diverticulum (CSD), the pregnancy sac is divided into three types^15^: Superficial: The basal decidua was partially attached to the caesarean incision scar without forming a diverticulum. Partial: The gestational sac was partially situated within the CSD. Complete: The gestational sac was entirely located within the CSD. The blood flow around the gestational sac (GS) at the site of the previous caesarean incision is categorized into four levels^16^: Grade 0: No blood flow signal detected. Grade I: Punctate blood flow observed in one to two locations. Grade II: One vessel longer than the radius of the lesion or several small vessels present. Grade III: More than four vessels or vessels interconnected forming a network. The foetal heartbeat status during gestation was categorized into two types based on ultrasound reports: absence of heartbeat and presence of heartbeat. Additionally, gestational sac diameter, the scar thickness between the gestational sac and bladder, and the presence of RPOC on ultrasound review one month postoperatively were also obtained.

**Clinical characteristics**The ages of all patients, along with their pregnancy histories, including the number of pregnancies, caesarean sections, induced abortions, and previous CSPs, were comprehensively examined and documented. Additionally, preoperative levels of β-hCG, intervals between surgery and ultrasound examinations, and surgical procedure details were meticulously reviewed and summarized.

### Construction and validation of the prediction model

A predictive model for the occurrence of complications during the management of CSP was established using the training set. The model underwent validation using both internal and external validation datasets. To address multicollinearity among different variables, the least absolute shrinkage and selection operator (LASSO) regression analysis was utilized to identify the most relevant predictive variables. Cross-validation was applied to ascertain the suitable tuning parameter (lambda) for the LASSO logistic regression. Subsequently, in the multivariate logistic regression analysis, a nomogram model was developed using variables with a significance level of *P* < 0.05. Calibration curves were then created to assess the accuracy of the adverse event nomogram in predicting outcomes. In the training set, the discriminative ability of the nomogram was evaluated using metrics such as the C-index and the area under the receiver operating characteristic curve (AUC). Additionally, decision curve analysis (DCA) was conducted to assess the practical utility of the nomogram. DCA evaluates net benefits by comparing the proportion of false-positive patients to true positive patients, while considering the potential adverse consequences of forgoing any unnecessary intervention, based on a predetermined threshold probability. The clinical impact curve analysis (CICA) categorizes patients into high risk or high risk with event and compares the actual observed event rates. The predictive accuracy of the nomogram was evaluated using various metrics on both the internal and external validation sets. This evaluation included the use of ROC curves, calibration curves, DCA, and CICA.

### Statistical analysis

Measurements that follow a normal distribution are presented as mean ± standard deviation (SD), while those that do not adhere to normal distribution are expressed as median (interquartile range). Categorical data are represented by counts or rates. The independent-sample t-test is used for comparing parameter values between two groups, while the Mann–Whitney U-test is used for comparing nonparametric values between two groups. The chi-square test is employed for comparing categorical variables. Lasso regression analyses and multivariate logistic regression analyses were conducted. All tests were two-tailed, and a significance level of *P* < 0.05 was considered statistically significant. Statistical analyses were carried out using the R statistical software package (version 4.3.0, Vienna, Austria) and GraphPad Prism (version 9.5.1, San Diego, CA, USA).

## Result

### Baseline characteristics of the included patients

**Fig. 1:**
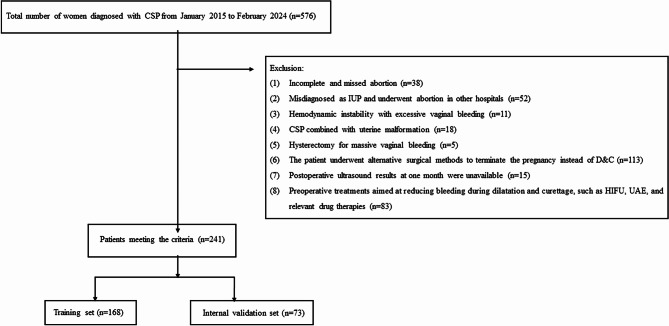
The flowchart of participants’ selection for the study in the internal cohort. CSP caesarean scar pregnancy, IUP intrauterine pregnancy, D&C dilation and curettage, HIFU high intensity focused ultrasound, UAE uterine artery embolization.

The flowchart depicted in Fig. 1 outlines the screening process for the internal cohort, which consisted of 241 eligible CSP patients participating in the study. This internal cohort was subsequently divided into a training set, comprising 168 individuals, and an internal validation set, comprising 73 individuals. Within this internal cohort, the case group, characterized by having experienced adverse events, comprised 80 out of the total 241 patients (33.20%), while the control group, without adverse events, comprised 161 out of 241 patients (66.80%). Moreover, an external cohort was selected using the same screening criteria, as shown in Supplementary Fig. 1. Table 1 displays the baseline characteristics of both the internal and external cohorts. Importantly, no statistically significant differences were observed between the case and control groups concerning age, menopausal duration, and the number of previous caesarean sections in the internal and external cohorts (*p* > 0.05).

**Table 1:** Patient demographics and preoperative characteristics.

Characteristics	Internal cohort (*n* = 241)			External cohort (*n* = 120)		
	Case group (*n* = 80)	Control group (*n* = 161)	*P* value	Case group (*n* = 40)	Control group (*n* = 80)	*P* value
**Age**,** years**	33.52 ± 4.60	33.25 ± 3.03	0.59	34.33 ± 4.37	34.51 ± 3.41	0.81
**Menopause (days)**	54.61 ± 10.11	54.97 ± 10.35	0.80	52.98 ± 8.54	52.23 ± 9.83	0.68
**Serum β-hCG (mIU/ml)**	55,451 ± 56,570	47,289 ± 59,299	0.31	38,195 ± 37,424	38,474 ± 37,180	0.97
**Gravidity**	3.80 ± 1.49	4.03 ± 1.82	0.33	3.49 ± 1.58	3.88 ± 1.63	0.20
**Number of cesarean sections**	1.46 ± 0.61	1.48 ± 0.57	0.88	1.42 ± 0.63	1.42 ± 0.55	0.98
**Number of artificial-abortion**	1.80 ± 1.31	2.13 ± 1.63	0.12	1.56 ± 1.16	2.04 ± 1.57	0.08
**Scar thickness(mm)**	3.07 ± 1.11	2.18 ± 0.98	< 0.01	2.44 ± 1.13	3.05 ± 1.09	< 0.01
**Percentages of grade III blood flow**	20/80(25.00%)	18/161(11.18%)	0.03	15/40(37.50%)	10/80(12.50%)	0.02
**Mean gestational sac diameter (mm)**	33.54 ± 15.44	27.31 ± 14.47	0.03	34.88 ± 14.66	27.34 ± 14.25	< 0.01
**Percentages of type III CSP**	33/80(41.25%)	34/161(21.11%)	0.02	19/40(47.50%)	16/80(20.00%)	0.04
**Presence of heartbeat**	29/80(36.25%)	53/161(32.92%)	0.61	18/40(45.00%)	31/80(38.75%)	0.51

β-hCG β Human Chorionic Gonadotropin, CSP Cesarean scar pregnancy.

### Selection of predictive model variables

**Fig. 2:**
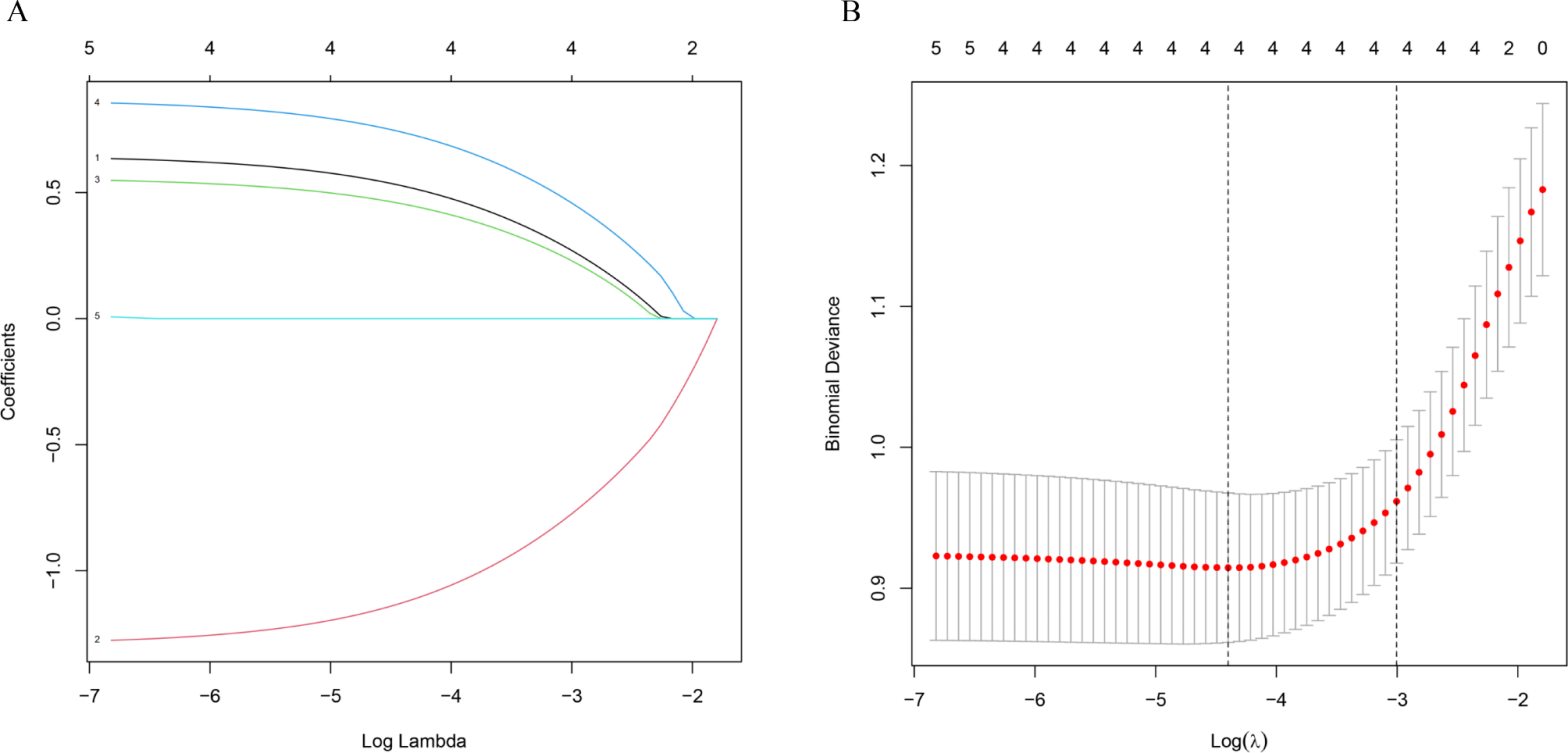
The process of predictor selection using the LASSO (Least Absolute Shrinkage and Selection Operator) regression method. (**A**) We observe the LASSO coefficient profiles of five variables plotted against the log(λ) sequence. (**B**) The selection of the best penalty coefficient lambda through tenfold cross-validation and minimization criterion. The optimal parameter (λ) in the LASSO model is verified by plotting the binomial deviance curve versus log(λ), with dotted vertical lines indicating the selected lambda. Four variables with nonzero coefficients were selected based on the optimal lambda.

In our analysis, we used LASSO regression to select variables, as shown in Fig. 2A. We noticed variations in coefficients for each variable. Furthermore, through tenfold cross-validation, we found an optimal model with superior performance and minimal variables, as depicted in Fig. 2B. The selected variables included gestational sac diameter, scar thickness, blood flow, CSP type, and presence of heartbeat. To develop a predictive model for adverse events in patients with CSP, we conducted a multivariate logistic regression analysis on the aforementioned five variables using LASSO regression technology. As shown in Table 2, four indicators were identified as independent risk factors influencing the occurrence of adverse events following D&C.

**Table 2:** Prediction variables for the risk of adverse events in CSP management.

Intercept and variable	Prediction model		
	β	Odds ratio (95% CI)	*P*-value
**Intercept**	-1.25	0.29 (0.14, 0.59)	< 0.01
**Gestational sac diameter**	0.41	1.51 (1.01, 2.27)	0.04
**Scar thickness**	-0.90	0.41(0.26, 0.61)	< 0.01
**Blood flow**	0.43	1.54(1.08, 2.21)	0.02
**CSP type**	0.60	1.81 (1.19, 2.80)	0.01

*β* is the regression coefficient, CI confidence interval.

### Construction and evaluation of the nomogram

**Fig. 3:**
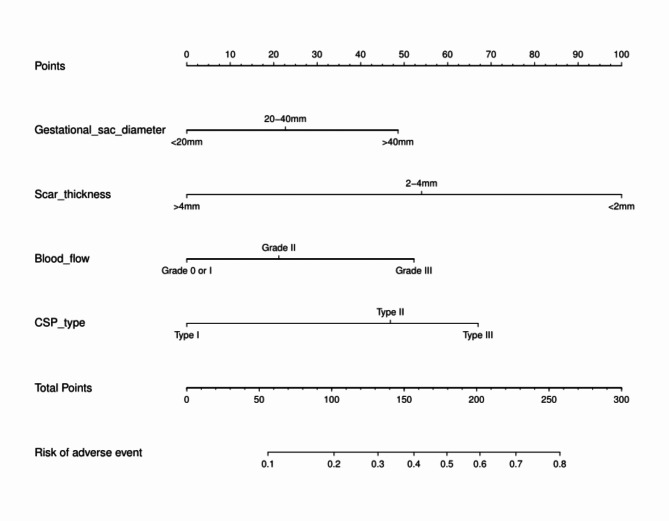
A nomogram to predict the risk of adverse events in CSP.

**Fig. 4:**
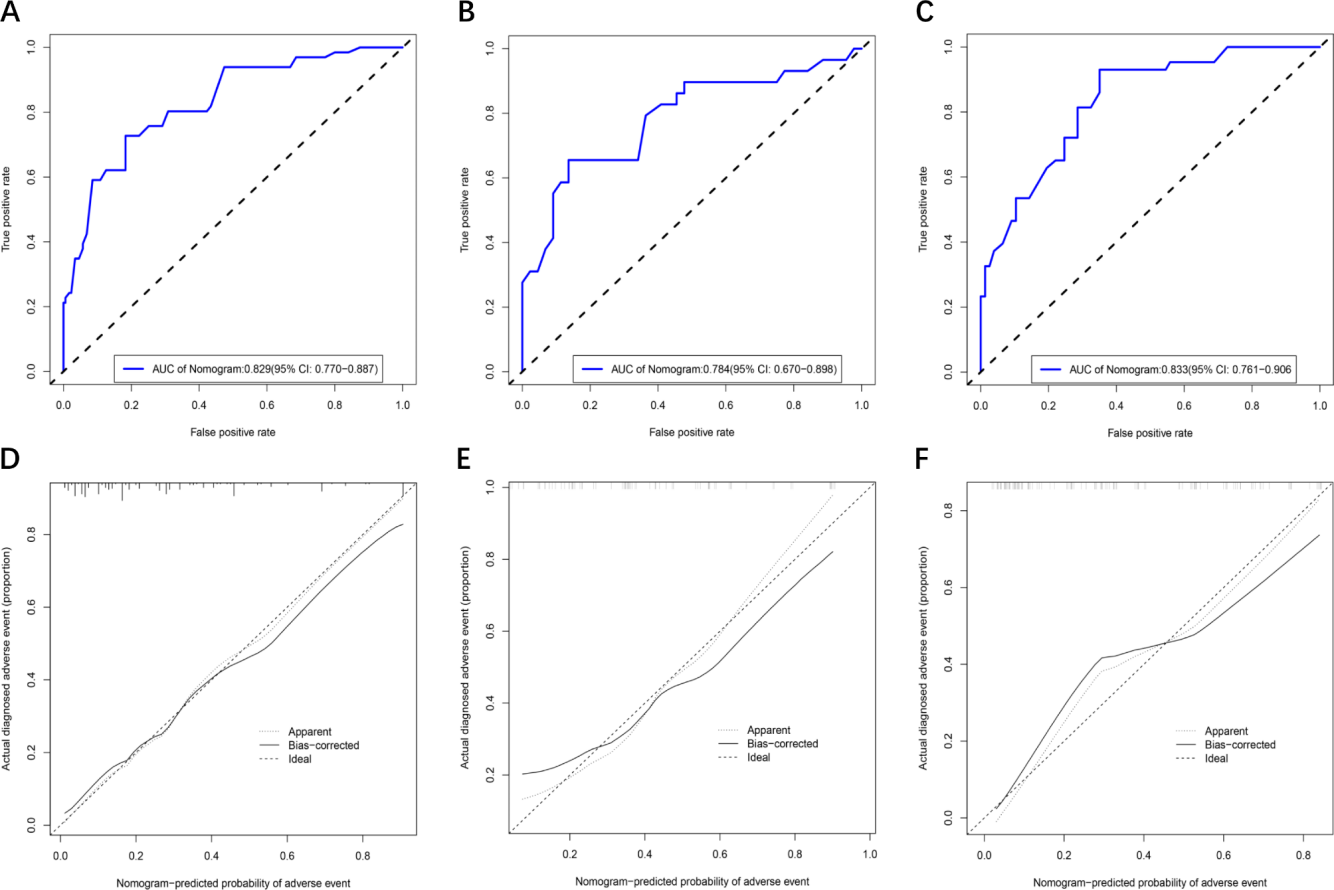
ROC curve and calibration curve of nomogram model in dataset of training set, internal validation set and external validation set, respectively. A-C for ROC curve and D-F for calibration curve.

**Fig. 5:**
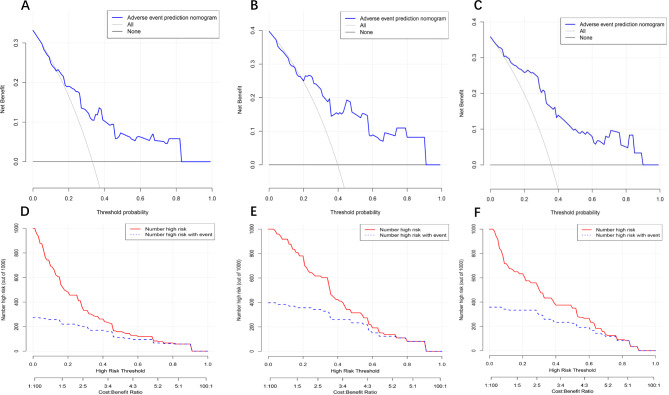
Decision curve analysis (DCA) and clinical impact curve analysis (CICA) of nomogram model in training set, internal validation set, and external validation set. A-C for DCA and D-F for CICA.

A nomogram was constructed based on the four predictive factors for adverse events identified in the training set (Fig. 3). This nomogram revealed that scar thickness had the most significant influence on the incidence of adverse events in patients with CSP, followed by CSP type, blood flow, and gestational sac diameter. The predictive model demonstrated good discriminative ability, with a C-index of 0.83 (95% CI: 0.77–0.89) for the training set (Fig. 4A). Furthermore, calibration curve analysis was performed to assess the degree of fit of the nomogram. The calibration curve exhibited a favourable agreement between prediction and observation in the training cohort (Fig. 4D). The Hosmer-Lemeshow test resulted in a P-value of 0.59, indicating that the model was well-fitted in the training set. Subsequently, the clinical utility of the prediction model was evaluated through DCA and CICA. In the training set, both DCA and CICA demonstrated that the nomogram provided greater overall net benefits across a broad and clinically relevant range of threshold probabilities, thereby influencing the clinical prognosis of patients. Therefore, while the nomogram demonstrates potential clinical value, this conclusion can only be confirmed through prospective testing and application in future CSP cases (Fig. 5A and D).

### Validation of nomogram

The study further validated the nomogram through an internal validation set and external validation set. The C-index for the prediction nomogram was 0.78 (95% CI: 0.67–0.90) in the internal validation set and 0.83 (95% CI: 0.76–0.91) in the external validation set, respectively (Fig. 4B and C). The calibration curve demonstrated strong consistency between predicted and observed risk of adverse events across the two validation sets (Fig. 4E and F). The DCA (Fig. 5B and C) and CICA (Fig. 5E and F) of both the two validation sets yielded excellent net clinical benefit.

## Discussion

Currently, research interest in CSP has shifted from image-based diagnosis to optimal management. Regarding CSP management, the primary focus is on selecting the best treatment modalities to reduce complication risks and preserve fertility. Surgical and non-surgical treatments are the main modalities for treating CSP. For different types and severities of CSP, choosing the appropriate surgical approach can significantly impact treatment outcomes. For example, Xu et al.^17^ adopted different measures for different types of CSP during lesion excision surgery to achieve optimal treatment outcomes. Additionally, reducing treatment complications through improved surgical techniques is also a focal point of CSP management. For instance, Ellen Hofgaard et al.^18^ achieved better clinical outcomes in treating CSP by employing robot-assisted laparoscopy. Preoperative interventions can also reduce surgical risks^19^. In our meta-analysis on CSP treatment^20^, it was found that preoperative UAE or HIFU can increase the success rate of surgery and significantly reduce postoperative complications after D&C. Long-term reproductive outcomes of CSP are also a focal point^21,22^, especially for patients with fertility requirements. In summary, before selecting the optimal treatment modality, a detailed assessment of CSP is necessary to achieve the best treatment outcomes and avoid serious complications.

In previous reports, most studies^12,23–25^ were single-centre, utilizing univariate and multivariate logistic regression analyses to identify clinical and/or imaging indicators for determining risk factors for massive bleeding or RPOC during CSP termination of pregnancy. In this study, we developed and validated, for the first time, a column chart-based lasso regression method to select ultrasound indicators for preoperatively predicting the risk of bleeding or RPOC during CSP curettage management. This nomogram incorporates four ultrasound measurements of CSP, including scar thickness, gestational sac diameter, and blood flow, as well as CSP type as relevant risk factors for CSP, to achieve personalized prediction of occurrence adverse events in CSP patients. The nomogram developed based on ultrasound indicators assists clinicians in identifying high-risk patients, such as those with intraoperative bleeding ≥ 200 ml or RPOC, thereby guiding the development of appropriate treatment plans. This includes selecting experienced specialists to perform UAE or local methotrexate injection to control blood supply to the gestational sac before surgery. If these measures are ineffective, surgical treatment may be necessary, including lesion removal, uterine repair, and, in severe cases, hysterectomy. Overall, complex cases require the collaboration of a multidisciplinary team, including obstetrics and gynaecology, interventional radiology, and anaesthesiology, to develop and implement personalized treatment plans.

Studies have reported^5^ that patients with CSP experience significantly higher levels of bleeding during D&C compared to those during miscarriage and abortion. This may be attributed to the attachment of the CSP gestational sac to the scar tissue of the uterine muscle layer, where the lack of decidua tissue makes it easier for trophoblastic cells to invade beyond the junction of the endometrium and uterine muscle layer, reaching the deep uterine blood supply from the radial and arcuate arteries^26^, leading to a rapid increase in blood flow around the gestational sac. Therefore, forcibly separating the gestational sac during D&C may result in uncontrolled bleeding due to the weak muscle layer’s inability to contract. Furthermore, as the gestational age increases, the pregnancy mass enlarges, potentially elongating and thinning the lower segment of the uterus. This combined with an adequate blood supply, can lead to increased bleeding during the surgical procedure. Therefore, it is believed that a thinner the uterine muscle layer in the anterior wall scar results in a more pronounced the increase in blood flow around the gestational sac site of the previous caesarean section incision, and the greater the likelihood of significant intraoperative bleeding.

The incidence of RPOC in patients with CSP is higher than that in those after miscarriage^5,27,28^. The possible reasons for the persistent residual mass of ectopic pregnancy are as follows: (1) The CSP mass enters the uterine muscle layer or scar depth through micro-fissures, such as in type III CSP, and may sometimes invade the broad ligament, making surgical excision difficult^29^; (2) In case of significant intraoperative bleeding before complete emptying of the pregnancy product, surgery must be stopped to ensure haemostasis; (3) Local bleeding occurs before the complete removal of the gestational sac; and (4) Scar tissue at the uterine incision site may hinder its complete removal. Additionally, CSP patients typically undergo multiple routine follow-up examinations after surgery, which helps to improve the diagnosis rate of RPOC. Time is also crucial for terminating CSP, as with time, the gestational sac and its blood vessels grow, increasing the difficulty of D&C and the likelihood of residual RPOC^4^. When trophoblastic tissue is situated at the scar site and the uterine muscle layer is thinner, there is an elevated risk of uterine perforation during D&C surgery. Additionally, a richer blood flow around the gestational sac increases the likelihood of bleeding during the procedure. These factors compound the surgical difficulty and may escalate the likelihood of RPOC. Consequently, for patients at a higher risk, personalized treatment plans such as hysteroscopy surgery or laparoscopic monitoring are preferable options.

There are several limitations to this study. Firstly, both the internal and external cohorts of this study come from different hospitals in the same province, limiting the external generalizability of the results. Secondly, this study is a retrospective cohort study, which cannot control for the consistency and completeness of data collection. Moreover, a prospective approach should be adopted to collect relevant data when validating the model’s effectiveness. Thirdly, the nomogram exclusively incorporated ultrasound-related indicators, which restricts the model’s practicality in patients with unclear ultrasound images.

## Conclusions

In summary, our study has yielded a predictive nomogram specifically designed to assess the risk of adverse outcomes in CSP patients. This nomogram exhibits strong discriminatory and calibration capabilities, offering promising prospects for enhancing clinical decision-making in the management of CSP. Its potential to improve risk assessment and guide personalized patient care underscores its significance as a valuable clinical tool in this context.

## Electronic supplementary material

Below is the link to the electronic supplementary material.

Supplementary Material 1

## Data Availability

The datasets used during the current study available from the corresponding author on reasonable request.
