# Supplementary figures and images for: Predictive nomogram of ultrasound indicators for the termination outcome of caesarean scar pregnancy
Source: Sci Rep. 2024 Dec 28;14:31378. doi: 10.1038/s41598-024-82894-7 (PMC11682213; doi:10.1038/s41598-024-82894-7)

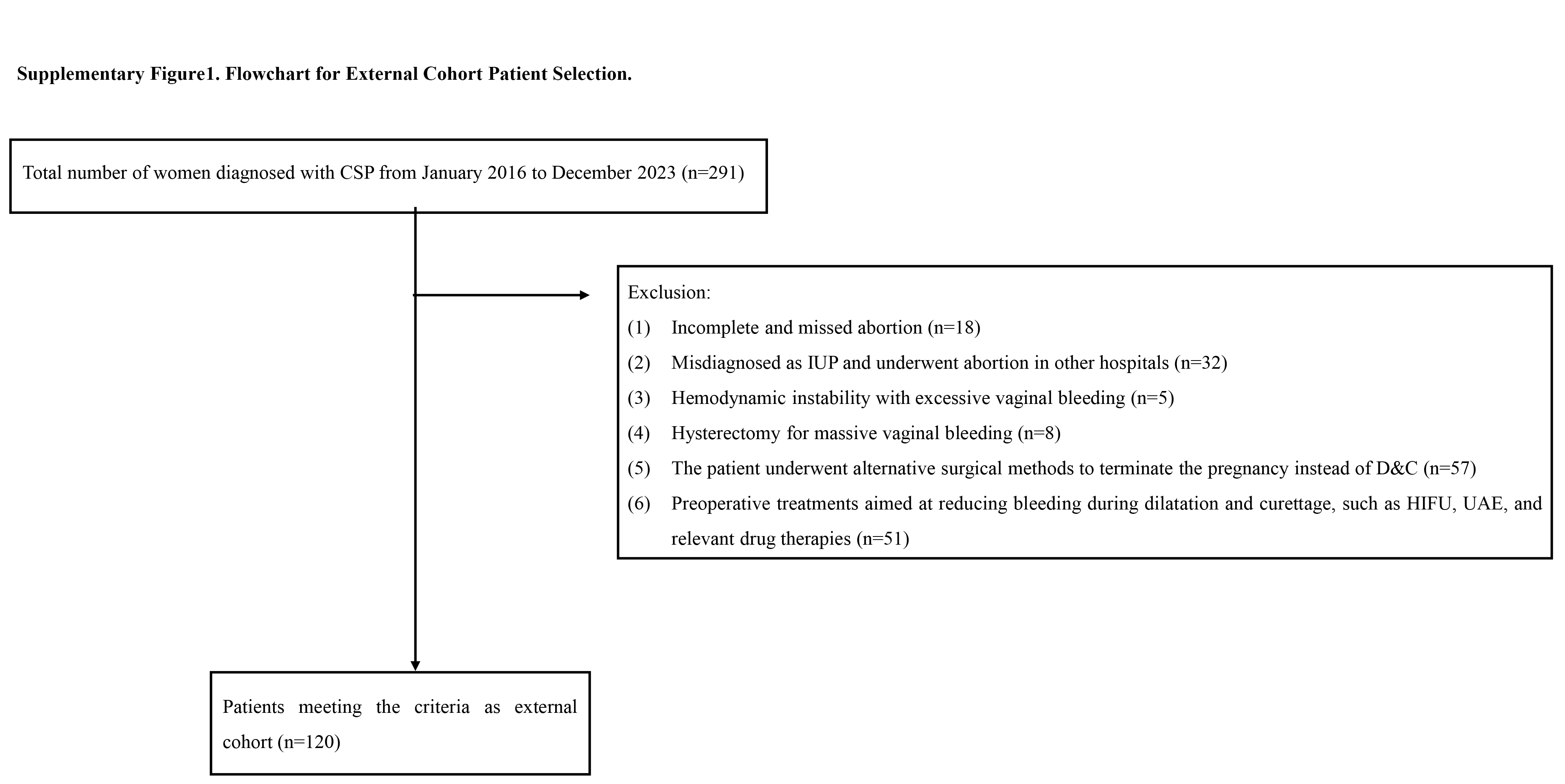

Supplement: Supplementary file 1 — Supplementary Material 1 [file 41598_2024_82894_MOESM1_ESM.tif]
